# MiR-144 promotes β-amyloid accumulation-induced cognitive impairments by targeting ADAM10 following traumatic brain injury

**DOI:** 10.18632/oncotarget.19469

**Published:** 2017-07-22

**Authors:** Liqian Sun, Manman Zhao, Jingbo Zhang, Aihua Liu, Wenjun Ji, Youxiang Li, Xinjian Yang, Zhongxue Wu

**Affiliations:** ^1^ Department of Interventional Neuroradiology, Beijing Neurosurgical Institute and Beijing Tiantan Hospital, Capital Medical University, Beijing 100050, P.R. China; ^2^ Department of Histology and Embryology, School of Basic Medical Science, North China University of Science and Technology, Tangshan 063000, P.R. China

**Keywords:** miR-144, ADAM10, TBI, cognitive impairment, β-amyloid

## Abstract

The dysregulation expression of microRNAs (miRNAs) including miR-144, has been widely documented in TBI. However, little is known about the potential roles of miR-144 in the pathogenesis of TBI. In this study, we investigated the potential effects of miR-144 on cognitive function *in vivo* and *in vitro*. The results indicated that inhibition of miR-144 conferred a better neurological outcome after TBI *in vivo*, as evidenced by reduced lesion volume, alleviated brain edema and increased mNSS, of particular importance, improved cognitive deficits. *In vitro*, miR-144 knockdown protected neuron against Glu-induced injury, by enhancing cell viability, suppressing LDH release and caspase-3 activity, and reducing cognitive-related proteins levels. However, overexpression of miR-144 *in vivo* and *in vitro* showed the opposite effects. To further explore the molecular mechanisms underlying miR-144-induced cognitive dysfunctions, we found a significant inverse correlation between miR-144 and ADAM10 expression. Moreover, the direct interaction between miR-144 and ADAM10 3’-UTR was identified by dual-luciferase reporter assay. Also, we found miR-144 negatively regulated ADAM10 protein expression. Additionally, ADAM10 could modulate β-amyloid formation involved in cognitive deficits. Notably, ADAM10 knockdown by siRNA apparently abrogated miR-144 inhibitor-mediated neuroprotection. Taken together, these findings demonstrated that elevated miR-144 promoted cognitive impairments induced by β-amyloid accumulation post-TBI through suppressing of ADAM10 expression.

## INTRODUCTION

Traumatic brain injury (TBI) is one of the death-related diseases with high morbidity and mortality, especially in children and young adults [1]. An estimated 10 million people worldwide suffer from TBI annually, and TBI will become the third most common cause of global disease burden by 2020 [2]. It initiates secondary cell death mechanisms that contribute to neurological dysfunction, such as motor and sensory dysfunction, and cognitive deficits in spatial learning and memory [3, 4]. The hippocampus is a key brain region for cognition, and is particularly vulnerable to injury [5]. TBI-related hippocampus damages, including neuron loss, impaired synaptic transmission and synaptic plasticity, is strongly related to cognitive impairments [6, 7]. There are a complex series of signaling molecules and pathways involved in cognitive deficits after TBI. It is well known that alteration of various genes are involved in pathophysiological processes of TBI [8]. More interestingly, epigenetic modifications, such as miRNAs dysregulation, may alter genes expression in the brain, especially hippocampus [9–13]. However, the precise molecular mechanisms underlying cognitive impairments following TBI remain largely unknown. Therefore, identification of the specific miRNAs and genes involved in TBI-mediated cognitive deficits is of great importance for developing novel therapeutic strategies for TBI.

MiRNAs, a class of endogenous small non-coding RNAs, could negatively regulate gene expression at the post-transcriptional level by binding to the 3’-UTR of target miRNAs, leading to their degradation and/or translational repression [14, 15]. It is estimated that mRNAs regulate more than 50% of human protein-coding genes [16]. Not surprisingly, the involvement of miRNAs in regulating various biological processes, including growth, development, differentiation and death, provides novel insights in disease research [17]. Additionally, some miRNAs exhibit phylogenetically conserved organ-specific and tissue-specific expression [18]. Sempere et al has identified a subset of brain-specific miRNAs, including miR-9, miR-124a/b, miR-135, miR-144, miR-153 and miR-183, whose expression behavior are conserved in both mouse and human differentiating neurons [19]. Suggesting miRNAs could play an important role in mammalian neuronal development/function and a variety of physiological processes in the central nervous system (CNS). For instance, miRNAs are implicated in dendriticspine development, regulated neurites outgrowth, maintained synaptic plasticity, controlled human neuronal differentiation, and promoted neurogenesis [20, 21].

Over the past several years, it has become clear that alterations of miRNAs expression could cause neurodegenerative disorders, including Alzheimer's disease (AD), Parkinson disease, Huntington disease, and ischemic stroke [22–24]. However, little attention has been paid to their roles in acute diseases for CNS, such as TBI. Like other diseases, the development of TBI is a multistep process with accumulation of genetic and epigenetic changes. Recently, Meissner L has investigated the temporal profile of miRNAs expression during the development of experimental TBI by microarray analysis, 9 miRNAs were significantly up-regulated (miR-21, miR-144, miR-184, miR-451, miR-2137) and down-regulated (miR-107, miR-137, miR-190, miR-541) in TBI compared with sham-operated mice [25]. Moreover, 10 miRNAs consistently up-/down-regulated have been reported at all five time points after TBI, among upregulation miRNAs in rat ipsilateral hippocampus also included miR-144 [10]. Meanwhile, a large number of target genes related to TBI pathophysiology in bioinformatics and gene ontology analyses were targeted by miR-144 and miR-340-5p [10, 25]. Recently, it is further demonstrated that strong positive correlations miR-144 and cognitive dysfunctions [26]. Although a number of miRNAs are associated with TBI have been identified to date, the involvement of miRNAs in TBI-induced pathophysiological alterations and the contribution of miRNAs to the TBI-mediated cognitive deficits remain to be elucidated. On the basis of these above findings, we mainly focused on expression and biological functions of miR-144, characterized by brain-specific expression, continuously elevated post-TBI, and closely related to TBI-induced cognitive function.

miR-144 was previously reported to be increased in the aging primate cerebellum, nonhuman primate cortex and AD patients [26–28]. It has been reported that the loss of miR-144 repressed proliferation and metastasis of glioblastoma (GBM) cells by targeting c-Met, which effectively predicted overall survival in glioma patients [29]. Furthermore, accumulative date demonstrated that the expression of miR-144 in brain microvascular endothelial cells plays a crucial role in regulation of blood-tumor barrier (BTB) permeability [30]. Notably, miR-144 appeared to be highly associated with the aging progression, neurodegenerative disorders and cognitive disorders by regulating the expression of ataxin 1 (ATXN1) at post transcription level [26]. Emerging evidence has shown the strongly association of aberrantly expressed miR-144 and neurological diseases [31]. However, the role of miR-144 in the development of TBI is not well documented.

In this study, the aberrantly expressed miR-144 in the plasma of TBI patients was first confirmed to be significantly up-regulated, which attracted our attention. Similarly, miR-144 also was determined to be dramatically increased in the rat model of TBI *in vivo* and neuron exposed to glutamate *in vitro*. Here, to further explore the functions and mechanisms of miR-144 in TBI, we mainly focused on the effects of miR-144 on cognitive outcome and related pathological changes. Moreover, we identified whether miR-144 negatively regulated ADAM10 expression by directly targeting to the 3′ UTR of ADAM10 mRNA, which is closely associated with β-amyloid induced cognitive deficits. However, this study will provide potential therapeutic targets for TBI-mediated cognitive impairments, and open a new avenue for TBI treatment by manipulating miRNAs levels.

## RESULTS

### Plasma miR-144 was up-regulated in TBI patients and correlated with clinical parameters

**Figure 1:**
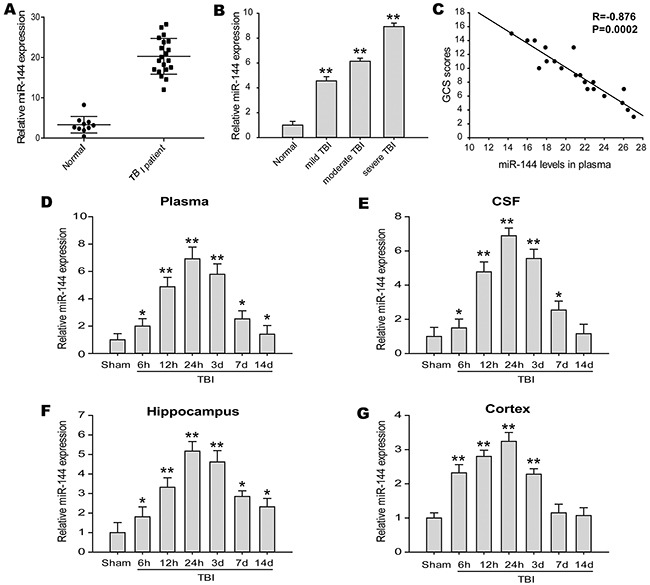
miR-144 expression was frequently up-regulated in TBI patients and experimental TBI rats **(A)** Plasma miR-144 levels were significantly increased in a large percentage of TBI patients (n=20) compared with healthy control subjects (n=10) detected by real-time PCR and normalized to the expression of U6. **(B)** The expresssion of miR-144 in the plasma of different degree of TBI patients. **(C)** Correlation between plasma miR-144 levels and GCS scores in TBI patients. **(D-G)** Relative expression of miR-144 were also markedly elevated in the plasma **(D)**, cerebrospinal fluid (CSF) **(E)**, hippocampus **(F)** and cortex **(G)** of experimental TBI rats at 6 h, 12 h, 24 h, 3 d, 7 d and 14 d post-injury. The data are represented as mean ± SD from three independent experiments. *P< 0.05, ^*^P< 0.01 versus the normal or sham group.

We detected the circulating level of miR-144 in the TBI patients and the healthy controls with real-time PCR. Compared to the healthy controls, the levels of miR-144 were significantly increased in the plasma from the patients with TBI (Figure 1A). A remarkable increase of miR-144 in plasma was first confirmed in this study. In addition, the Glasgow Coma Scale (GCS) was employed to determine the neurological deficits after TBI to estimate the severity of brain injury. 13-15 GCS scores considered mild TBI, 9-12 GCS scores considered moderate TBI, and 3-8 GCS scores considered severe TBI. Next, we evaluated alterations of miR-144 expression in different severity of TBI patients, and found miR-144 levels were markedly up-regulated in severe TBI patients relative to moderate or mild subjects (Figure 1B), indicating that the higher level of miR-144 was positively correlated with the severity of brain injury. The correlation between miR-144 level and GCS scores was analyzed (Figure 1C). A negative correlation existed between miR-144 level and GCS scores. Thus, these results demonstrated that the level of miR-144 is clinically significant in the context of TBI.

### miR-144 was increased in the experimental TBI rats

**Figure 2:**
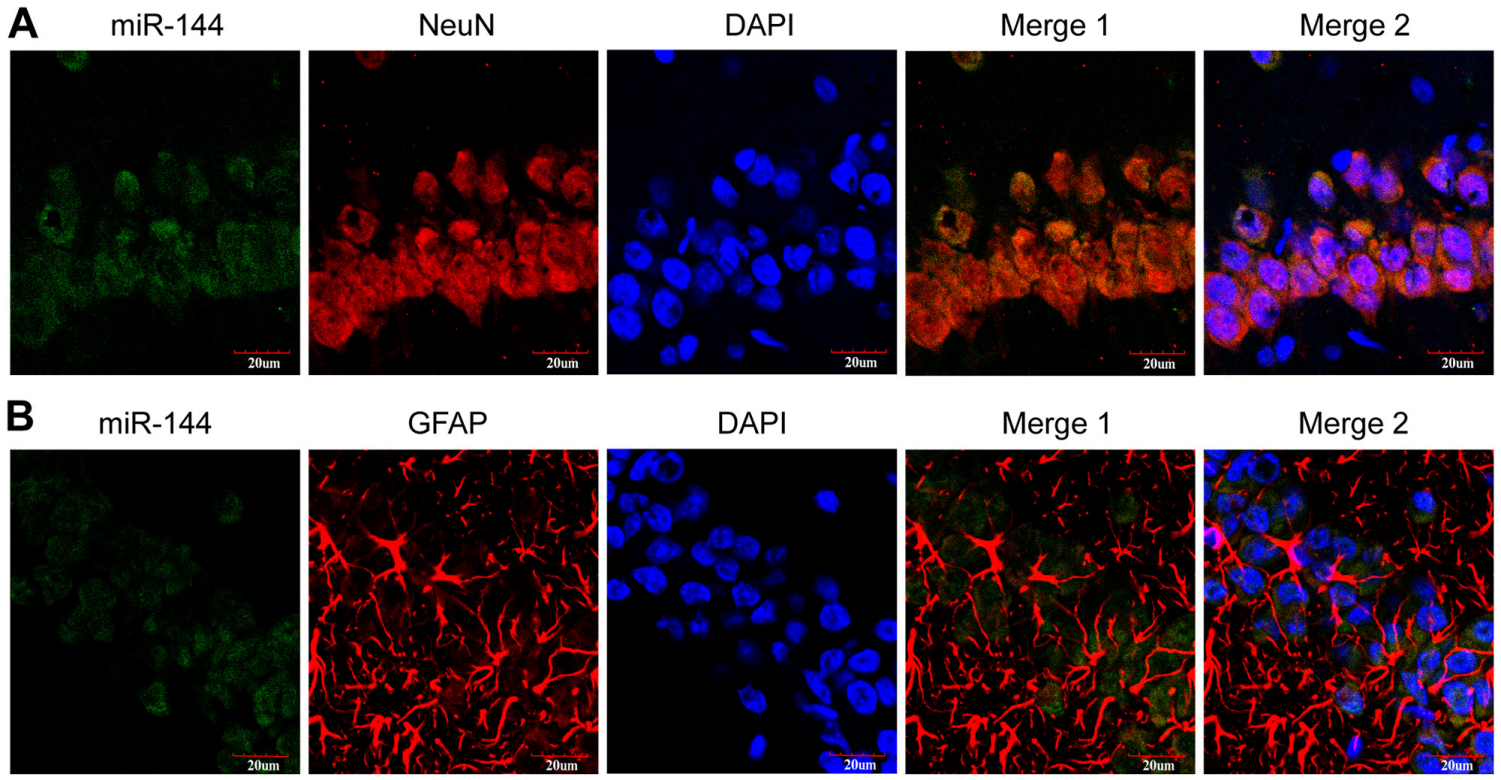
Representative images of the immunostaining of in-situ miR-144 expressed by neurons **(A)** and astrocytes **(B)** in the hippocampus CA1 region. These were measured by immunofluorescence staining and observed under the confocal laser scanning microscope. Scale bars: 20 mm.

We further examined miR-144 expression of the blood and cerebrospinal fluid (CSF) samples in the rat model of TBI, at different time-points from 6 h-14 d post-trauma. The date showed miR-144 expressions were up-regulated in the plasma and CSF of TBI rats when compared with the sham group (Figure 1D and 1E). The relative expression levels of miR-144 increased from 6 h post-injury, reached the peak at 24 h post-injury, and then decreased gradually. To further investigate whether the increase of miR-144 has regional and spatial specificity in brain tissue. In previous animal models, the expression miR-144 in the cortex and hippocampus were examined with real-time PCR. The results indicated that miR-144 expression was significantly increased in the hippocampus (Figure 1F) and the cortex (Figure 1G) after TBI compared with that in sham group. Whereas, the upregulation of miR-144 in the hippocampus was more significant than in the cortex. The level of miR-144 in the hippocampus was up-regulated 6 h post-injury, peaked at 24 h post-injury and then gradually declined at 3-14 d post-TBI. The expression pattern of miR-144 was consistent with previous reports [10, 19]. Additionally, we observed the in-situ expression of miR-144 in the neurons and astrocytes at 24 h post-TBI using combined miRNA in-situ hybridization (ISH) and immunofluorescence (IF) staining (Figure 2)

### Inhibition of miR-144 protected rats against TBI-induced brain injury

**Figure 3:**
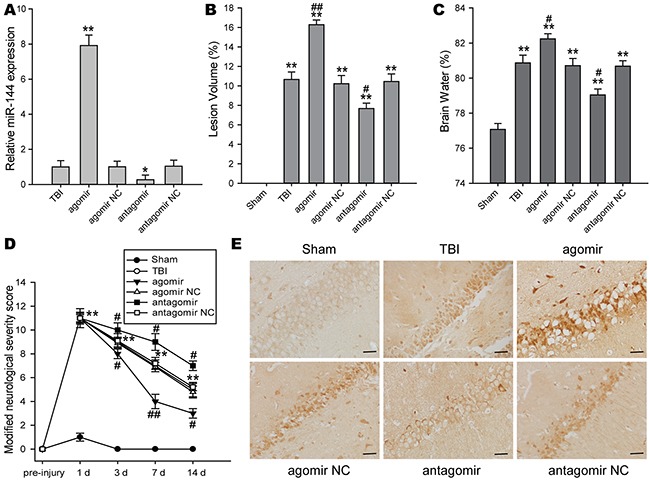
The impacts of regulating brain miR-144 on brain injury and neurological deficits after TBI *in vivo* **(A)** Plasma miR-144 levels were increased in the miR-144 agomir group and decreased in the miR-144 antagomir group at 48 h post-TBI. miR-144 agomir, antagomir and their corresponding NC were injected by the lateral ventricle at 15 min post-TBI. **(B-C)** Effects of treatment with miR-144 agomir or antagomir on cerebral lesion volume **(B)** and brain edema **(C)** at 3 d post-trauma. **(D)** The long-term neurological function was assayed by mNSS test at 1 d, 3 d, 7 d and 14 d after TBI. **(E)** Representative images of immunohistochemical staining of caspase 3 protein. Bar, 20μm. The data are represented as mean ± SD from three independent experiments. *P< 0.05, ^*^P< 0.01 versus the sham group, ^#^*P*< 0.05, ^##^*P*< 0.01 versus the TBI group.

To investigate the roles of miR-144 in exprimental TBI rats, miR-144 agomir, antagomir, and their negative control were respectively injected into the lateral ventricle of rats following TBI. 48 h after the injection, the transfection efficiency of miR-144 in the hippocampus of rats was confirmed using real-time PCR (Figure 3A). The protective effects of miR-144 on brain injury were also evaluated by assessment of cortical lesion volume, brain edema, and the modified neurological severity score(mNSS) at different time-points after TBI, which were a surrogate marker for the histopathological outcome that impacted the cognitive dysfunction. The lesion volume and brain edema was reduced in TBI rats treated with miR-144 antagomir, whereas miR-144 agomir administration aggravated lesion volume and brain edema as compared to untreated TBI rats (Figure 3B and 3C). For the mNSS test, no difference in the neurological score was observed in rats injected with miR-144 agomir and antagomir at 1 d post-TBI. Compared with TBI group, mNSS score was increased in the miR-144 antagomir group and decreased in the miR-144 agomir group at 3 d, 7 d and 14 d post-TBI (Figure 3D). In addition, neuronal damage of hippocampus after TBI were assessed by immunohistochemical staining of apoptotic marker casepase 3. The result showed that miR-144 agomir aggravated neuronal death, whereas miR-144 antagomir attenuated neuronal injury (Figure 3E). Taken together, these results demonstrated that inhibition of miR-144 could decrease lesion volume, alleviate brain edema, and improve neurological deficit and histological outcome, suggesting effective neuroprotection of miR-144 knockdown against brain injury in TBI rats.

### Inhibition of miR-144 improved TBI-induced cognitive impairment

**Figure 4:**
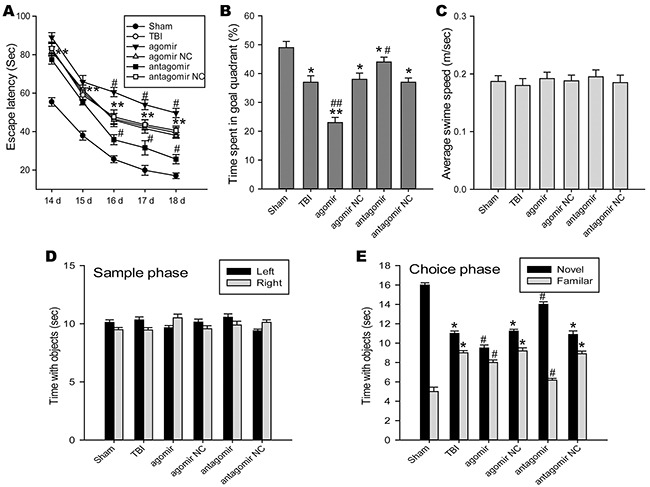
The roles of regulating brain miR-144 in cognitive deficits of TBI rats The cognitive function was evaluated by morris water mass (MWM) test including the spatial acquisition trial and the probe trial at 14 -18 d post-TBI, as well as novel object recognition (NOR) test at 18 d post-injury. **(A)** MWM performance as measured by the latency to find the hidden platform in the spatial acquisition trial (n=6). miR-144 antagomir treated group has significantly shorter latencies to reach the goal platform as compared to the TBI group. **(B)** Percentages of time spent in the goal quadrant during the probe trial (n=6). The rats administrated with miR-144 antagomir had significantly better retention of the platform location compared with rats suffered from TBI. **(C)** The overall average swim speed during test duration in different groups (n=6). **(D-E)** Intracerebroventricular injection of miR-144 antagomir improves retention memory using the NOR test at 18 d post-TBI (n=6). Following 24 h after the sample phase, the time spent with the novel object sand familiar objects during the choice phase was recorded. The data are represented as mean ± SD. *P< 0.05, ^*^P< 0.01 versus the sham group, ^#^*P*< 0.05, ^##^*P*< 0.01 versus the TBI group.

Spatial cognitive deficits was frequent comorbidities following TBI. Morris water maze (MWM) test was use to detect the changes of spatial learning and memory ability. Escape latency, the capability to navigate from a start location to a submerged platform, gradually reduced from 14 d to 18 d post-TBI. A repeated measures ANOVA showed escape latency was improved in the miR-144 antagomir group and impaired in the miR-144 agomir group as compared to the TBI group at 14 d to 18 d post-trauma (Figure 4A). The object of the probe trial was to assess retrograde reference memory. Compared with the TBI group, the miR-144 antagomir group displayed an increase in the average percentage of time spent in the target quadrant, whereas a decrease in the miR-144 agomir group (Figure 4B). No significant difference was revealed in average swim speed among the groups (Figure 4C), which revealed that the different performance was not due to motor impairments. To further examine the role of miR-144 on non-spatial hippocampus-mediated memory, novel object recognition (NOR) test was carried out at 18 d post-injury. We found the rats in the sham group spent more time than chance (10 s) with the novel object 1 day after training (choice phase), indicating intact memory. Notably, TBI rats transfected with miR-144 antagomir spent significantly more time with the novel object when compared with the TBI group, while miR-144 agomir-treated TBI rats spent less time with the novel object (Figure 4D and 4E). Collectively, these date demonstrated that higher level of miR-144 post-injury could promote TBI-induced cognitive deficits, and Inhibition of miR-144 protected TBI rats against cognitive and momery disorders.

### Inhibition of miR-144 prevented TBI-induced LTP impairment

**Figure 5:**
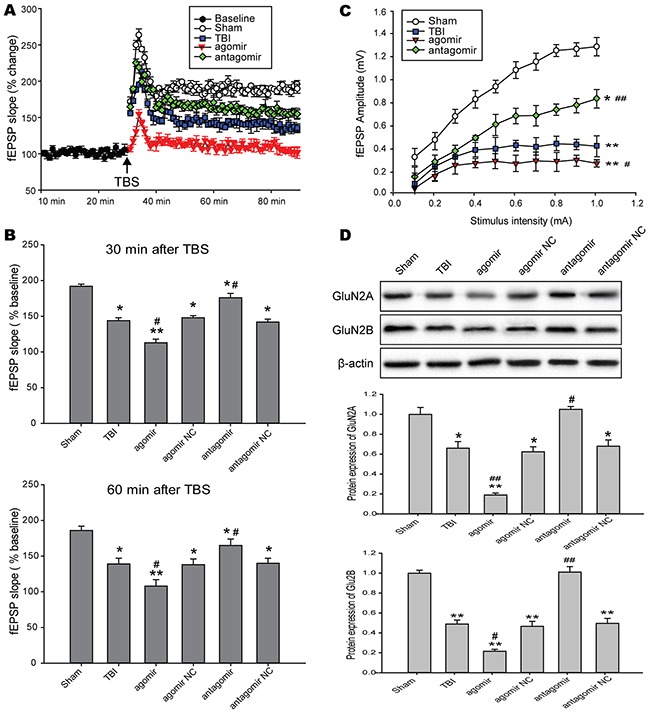
Effects of regulating brain miR-144 on LTP in NMDAR-dependent in rat hippocampal slices following TBI **(A)** The slope values of fEPSPs were analyzed for quantization of the responses. After 30 min of baseline recording of fEPSPs, TBS was applied and then the responses were measured for 60 min after TBS. **(B)** Histograme representing the average percentage LTP calculated using slopes of evoked responses at 30 min and 60 min after LTP induction. **(C)** The input-output (I-O) curves by the stimulation current intensity adjusted from 0 to 1 mA. **(D)**. Representative bands and quantitative analysis of NMDAR subunits in the hippocampal CA1 region of TBI rats in each group. The data are expressed as mean ± SD. *P< 0.05, ^*^P< 0.01 versus the sham group, ^#^*P*< 0.05, ^##^*P*< 0.01 versus the TBI group.

After stabilizing the baseline fEPSP recording at layer 2/3, TBS was applied to layer 4 to induce long-term potentiation (LTP) in the CA1 region of the hippocampus. The extent of LTP, calculated by averaging the slope values of fEPSPs recorded at 60 min after TBS. The results suggested that there was a significant increase in the degree of LTP in rats subjected to miR-144 antagomir and a decrease in rats treated with miR-144 agomir compared with TBI rats (Figure 5A). Meanwhile, we calculated the initial and final the slope values of field potentials at 30 min and 60 min after LTP inducted by TBS. miR-144 agomir treatment suppressed LTP, but miR-144 antagomir injection reinstated LTP post-TBI at 30 min and 60 min after LTP inducted by TBS (Figure 5B). Additionally, we generated input-output (I-O) curves by the stimulation current intensity adjusted from 0 to 1 mA. The data showed that miR-144 agomir significantly reduced fEPSP amplitude, whereas miR-144 antagomir notably up-regulated fEPSP amplitude (Figure 5C). This burst-dependent plasticity depended on postsynaptic N-methyl-D-aspartate receptor (NMDAR), and NMDAR is critically involved in hippocampus-dependent learning, hippocampal synaptic plasticity and memory encoding [32]. NMDAR typically comprise two subunits both GluN1 and GluN2 (including NR2A and NR2B). The hippocampal neurons expressed amounts of NR2A and NR2B subunits that was strongly related to learning and momery. Western blot analysis suggested that the levels of NR2A and NR2B were dramatically reduced in the hippocampus CA1 region of TBI rats transfected with miR-144 agomir and significantly up-regulated in TBI rats injected with miR-144 antagomir compared with the TBI group (Figure 5D). Taken together, these findings indicated that repression of miR-144 could control the maintenance of hippocampus LTP in an NMDAR-dependent manner and long-term memories over time.

### Expression of miR-144 was increased in Glu-induced neuron injury *in vitro*

**Figure 6:**
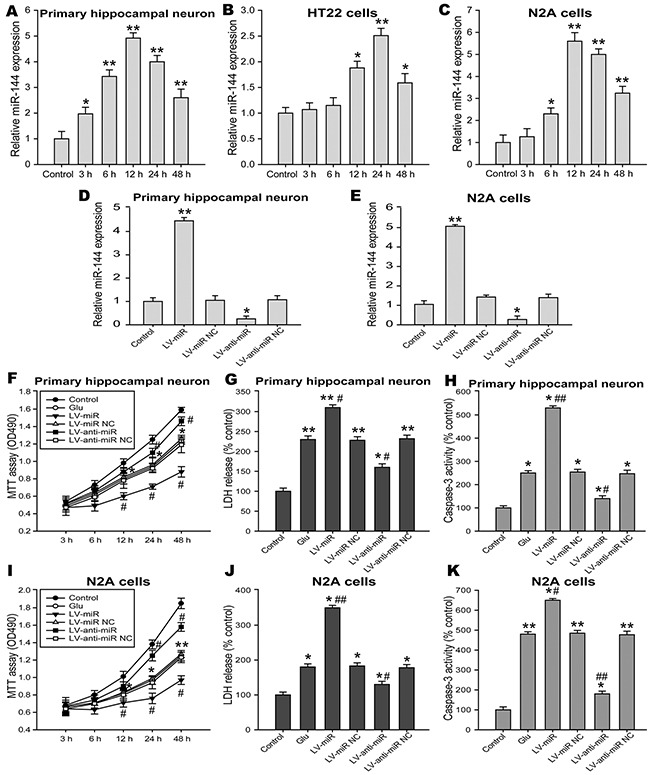
Effects of miR-144 on glutamate-induced neuron injury *in vitro* **(A-C)** Ectopic expression of miR-144 in primary hippocampal neuron, HT22 and N2A cells exposed to glutamate detected by real-time PCR and normalized to the expression of U6. **(D, H)** The infection efficiency of hippocampal neuron and N2A cells was determined by real-time PCR. **(E-G)** Effects of regulating cell miR-144 on cell viability **(E)**, LDH release in the culture supernatant **(F)** and cell apoptosis **(G)** in primary hippocampal neuron. Cell viability, LDH release and cell apoptosis were evaluated by MTT assay, LDH Kit and caspase-3 activity respectively. **(I-K)** Effects of regulating cell miR-144 on cell viability **(I)**, LDH release **(J)** and cell apoptosis **(K)** in the N2A cells. The data are represented as mean ± SD from three independent experiments. *P< 0.05, ^*^P< 0.01 versus the control group, ^#^*P*< 0.05, ^##^*P*< 0.01 versus the glutamate group.

Glutamate plays important roles as the predominant excitatory neurotransmitter in the mammalian brain. However, excessive release of glutamate results in excitotoxicity and is a major factor in neuronal injury associated with many acute and chronic brain disorders. Glutamate and glutamate transporter-1(GLT-1) play a crucial role in cognitive impairment caused by brain injury [33, 34]. As was indicated in Table 1, the level of glutamate were significantly increased in the damaged brain injury after TBI from 6 h to14 d. To explore the putative molecular mechanisms of miRNA-144-induced cognitive deficits, we employed an *in vitro* model of Glu-induced neuron injury, including primary hippocampal neurons, HT22 and N2A cells. Levels of miR-144 were analyzed by real-time PCR at various times after glutamate treatment. The results revealed that miR-144 expression significantly up-regulated in primary hippocampal neurons challenged to glutamate, peaked at 12 h post-injury (Figure 6A). Moreover, in the HT22 cells, we found miR-144 levels was not changed 3 h and 6 h post-injury, and then significantly increased, up to maximum at 24 h post-injury (Figure 6B). In addition, we observed similar patterns of miR-144 expression in the N2A cells challenged to glutamate (Figure 6C). Furthermore, miR-144 level in the N2A cells was higher than that in the HT22 cells.

**Table 1:** The level of glutamate in the hippocampus and cortex post-TBI (mean±SD, μmol/L)

Group	n	Hippocampus	Cortex
Sham	6	68.62±5.45	76.23±6.01
TBI 6 h	6	88.41±6.37*	82.91±7.22*
TBI 12 h	6	111.63±6.72*	102.56±5.78*
TBI 24 h	6	135.91±8.24*	120.36±7.16*
TBI 3 d	6	151.26±7.73*	142.97±6.85*
TBI 7 d	6	125.83±8.35*	118.34±7.41*
TBI 14 d	6	103.44±7.26*	98.53±6.74*

Note: *P< 0.05 versus the sham group.

### Inhibition of miR-144 attenuated Glu-induced neuron injury *in vitro*

To further confirm the functional significance of miR-144 in Glu-induced neuron injury *in vitro*. The primary hippocampal neurons and the N2A cells pretreatmented with lenti-miR-144, lenti-anti-miR-144 and their corresponding negative control respectively, were subjected to cell injury induced by glutamate. Forty-eight hours after transfection, real-time PCR analysis revealed that the levels of miR-144 notably increased in neurons transfected with lenti-miR-144, reversely, significantly decreased in neurons transfected with lenti-anti-miR-144 compared with untransfected ones (Figure 6D and 6E). Additionally, The cell viability (Figure 6F and 6I), LDH release (Figure 6G and 6J) and cell apoptosis (Figure 6H and 6K) were detected to evaluate the degree of cells damage. The results showed inhibition of miR-144 increased cell viability, suppressed LDH release and caspase-3 activity in the primary hippocampal neurons and the N2A cells. On the other hand, overexpression of miR-144 reduced cell viability, increased LDH release and caspase-3 activity. These results demonstrated the effective neuroprotective effects of miR-144 knockdown against Glu-induced neuron injury.

### Inhibition of miR-144 reduced cognitive-related proteins expression in neurons exposed to glutamate *in vitro*

**Figure 7:**
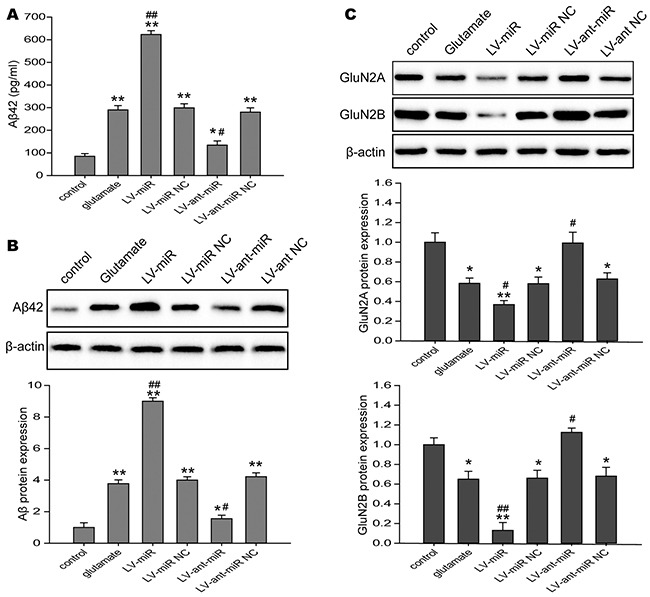
The impacts of regulating cell miR-144 on cognitive-related proteins in the N2A cells **(A)** The levels of Aβ_42_ in the culture measured using ELISA in N2A cells transfected with LV-miR-144 and LV-anti-miR-144. **(B)** The Western blots analysis of Aβ_42_ protein levels in N2A cells. β-actin served as an internal control. **(C)** Representive bands and quantitative analysis of NMDAR subunits in the N2A cells. The data are represented as mean ± SD. *P< 0.05, ^*^P< 0.01 versus the control group, ^#^*P*< 0.05, ^##^*P*< 0.01 versus the glutamate group.

The accumulation of β-amyloid (Aβ) in the brain induced synaptic damage, which is believed to be the essential cause of cognitive impairment [35, 36]. Moreover, NMDAR, synaptic plasticity-related molecule, is associated long-term memory and cognitive function [37]. Specially, β-amyloid peptides was reported may be modulate NMDAR expression [38]. Here, we investigated the levels Aβ_42_ and NMDAR in the N2A cells challenged to glutamate, and found that Aβ_42_ levels in the culture supernatant (Figure 7A) and the protein expression of Aβ_42_ (Figure 7B) in the N2A cells were up-regulated when miR-144 was overexpressed, but down-regulated when miR-144 was inhibited. In addition, the protein expression of NR2A and NR2B were significantly decreased in the N2A cells infected with lenti-miR-144, reversely, increased in the N2A cells treated with lenti-anti-miR-144 (Figure 7C).

### ADAM10 is direct target of miR-144

**Figure 8:**
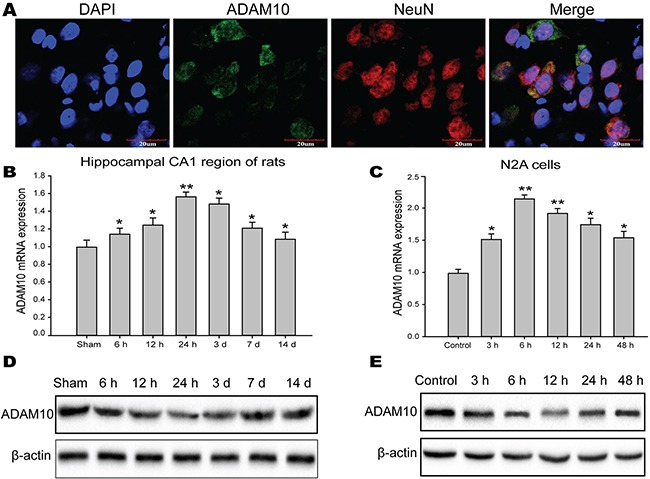
The protein level of ADAM10 was inversely correlated with miR-144 level **(A)** The localization and expression ADAM10 protein in the neuron of the rat hippocampal CA1 region. These were measured by immunofluorescence staining and observed under the confocal laser scanning microscope. **(B-C)** The mRNA level of ADAM10 in the hippocampal CA1 region of rats **(B)** and the N2A cells **(C)** measured by real-time PCR analysis. **(D-E)** The protein expression ADAM10 in the hippocampal CA1 region of rats **(D)** and the N2A cells **(E)** measured by Western blot. The data are showed as mean ± SD from three independent experiments. *P< 0.05, ^*^P< 0.01 versus the control group, ^#^*P*< 0.05, ^##^*P*< 0.01 versus the glutamate group.

**Figure 9:**
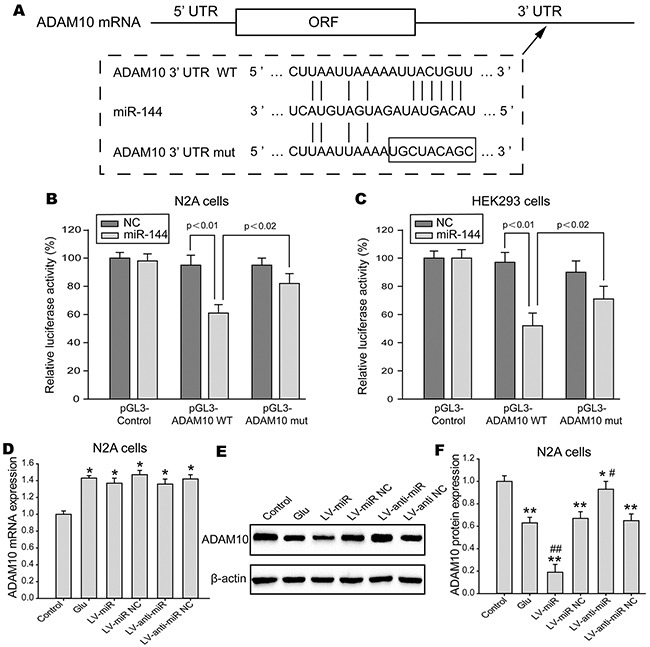
ADAM10 was direct downstream target for miR-144 **(A)** Schematic diagram of the miR-144 putative binding sites and corresponding mutant sites of the ADAM10 mRNA 3’-UTR. **(B-C)** The interaction between miR-144 and 3’-UTR of ADAM10 mRNA was detected relative luciferase activity assay in the N2A cells **(B)** and the HEK293 cells **(C)**. The WT or mut ADAM10 3’-UTR was co-transfected with LV-miR-144 and negative control. The luciferase activity was significantly decreased in cells co-transfected with WT ADAM10 3’-UTR and LV-miR-144, but not in cells co-transfected with mut ADAM10 3’-UTR and LV-miR-144. **(D-F)** Real-time PCR and Western blot analysis to quantify the mRNA and protein levels of ADAM10 in the N2A cells. Each experiment was performed at least three times, and each sample was analyzed in triplicate. The data presented mean ± SD.*P< 0.05, ^*^P< 0.01 versus the control group, ^#^*P*< 0.05, ^##^*P*< 0.01 versus the glutamate group.

To elucidate the molecular mechanisms underlying the functions of miR-144 in TBI, we employed TargetScan, miRanda, PicTar, and microRNA.org to identify candidate targets for miR-144 in genes involved in cognitive function. The gene for ADAM10, predicted by databases, was further studied as a potential target. First, immunostaining demonstrated that ADAM10 mainly located and expressed in hippocampal neuron (Figure 8A). Next, we analyzed ADAM10 protein and mRNA expression *in vitro* and *in vivo* studies. The data suggested that ADAM10 mRNA level was notably up-regulated in the hippocampus of TBI rats (Figure 8B) and the N2A cells exposed to glutamate (Figure 8C). However, the protein expression of ADAM10 was significantly decreased in rats hippocampal tissue post-TBI (Figure 8D) and the N2A cells post-injury (Figure 8E), and inversely correlated with endogenous levels of miR-144. This expression pattern of ADAM10 indicated miR-144 might inhibit ADAM10 expression by translational suppression. Furthermore, we found that miR-144 had conserved putative binding sites for ADAM10 mRNA on 3’-UTR (Figure 8A). To validate whether miR-144 directly binds to the 3’-UTR of ADAM10 mRNA and causes translational inhibition, using the dual luciferase gene reporter assay. The wild type (WT) 3’-UTR or the mutant (mut) 3’-UTR target sequences (Figure 9A) were cloned to luciferase reporter vector pGL3, and co-transfected with lenti-miR-144 or its negative control into the N2A cells or the HEK293 cells. The transfection of the WT pGL3- ADAM10-3′-UTR in combination with LV-miR-144 dramatically reduced the luciferase activity, and there was little activity when LV-miR-144 only was present. Moreover, mutation of the putative miR-144 binding sites clearly abrogated the suppression of luciferase activity caused by miR-144 overexpression in the N2A cells (Figure 9B) or the HEK293 cells (Figure 9C). Thus, these results indicated miR-144 directly bound to ADAM10 3’-UTR to inhibit ADAM10 expression at the post-transcriptional level.

To further verify the biological roles of miR-144 in regulating ADAM10 expression, the mRNA and protein levels of ADAM10 were quantified in gain and loss-function model of miR-144 *in vitro*. As expected, the results revealed that the protein level of ADAM10 was markedly decreased by miR-144 overexpression, in contrast, significantly increased by miR-144 inhibition in the N2A cells (Figure 9E and 9F). Whereas no significant change was observed in the mRNA levels of ADAM10 (Figure 9D). Suggesting that miR-144 negatively regulated ADAM10 expression by translational repressing rather than mRNA degradation. Thus, these results indicated that miR-144 could directly binding to ADAM10 3’-UTR to negatively regulate ADAM10 protein expression through promoting degradation of ADAM10 mRNA.

### ADAM10 is involved in cognitive deficits by modulating Aβ deposition

**Figure 10:**
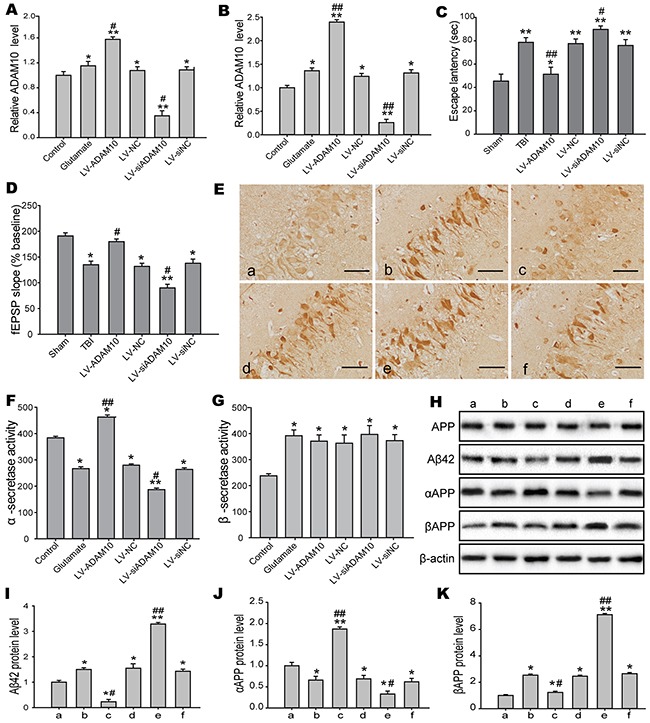
ADAM10 modulated the production of the Aβ peptides involved in cognitive impairments **(A-B)** Real-time RCR analysis was performed to assess the overexpresion or knockdown efficiency in the hippocampal CA1 region of rats **(A)** and the N2A cells **(B)**. **(C-D)** Effects of gain and loss of ADAM10 on TBI-induced cognitive dysfunction, including the hippocampus-dependent spatial learning and memory measured by MWM **(C)** and LTP recorded by electrophysiology **(D)**. **(E)** Representive images of Aβ_42_ deposition in the rat hippocampal CA1 region by immunohistochemical staining at 24 h post-injury. Bar, 20μm. **(F-G)** The α-secretase and β-secretase activity in the culture medium of the N2A cells using an activity assay at 12 h post-injury. **(H)** Western blot analysis of APP, Aβ_42_, αAPP, and βAPP protein expression levels in the N2A cells. **(I-K)** Densitometric analysis of Aβ42 **(I)**, αAPP **(J)**, and βAPP **(K)** protein with bands corresponding to β-actin. a: sham, b: TBI, c: LV-ADAM10, d: LV-NC, e: LV-siADAM10; f: LV-si NC. Data are expressed as mean ± SD. *P< 0.05, ^*^P< 0.01 versus the sham or control group, ^#^*P*< 0.05, ^##^*P*< 0.01 versus the TBI or glutamate group.

To identify the underlying mechanisms of ADAM10 for cognitive deficits in the present study, we induced and silenced ADAM10 expression *in vitro* and *in vivo*. Overexpression and knockdown efficiency was examined by real-time PCR in the hippocampus post-TBI (Figure 10A) and the N2A cells post-injury (Figure 10B). MWM test demonstrated that overexpression of ADAM10 markedly improved cognitive dysfunction, whereas silencing of ADAM10 significantly aggravated cognitive deficits after TBI (Figure 10C). Similarly, LTP induced by TBS was inhibited by ADAM10 knockdown but facilitated by ADAM10 overexpression following TBI (Figure 10D). These results showed that the decrease in ADAM10 induced by elevated miR-144 post-TBI, played a key role in cognitive deficits. Also, we detected some cognitive-related proteins levels *in vitro* and *in vivo*. The accumulation of Aβ_42_ peptide in the hippocampus CA1 of rats was showed in the immunohistochemical staining analysis. The result revealed that Aβ_42_ mainly deposited in the neurons of the hippocampus CA1 region. Moreover, Aβ_42_ deposition in the hippocampus CA1 was reduced in the LV-ADAM10 group, whereas aggravated in the LV-siADAM10 group (Figure 10E). Meanwhile, to address the potential of rats to produce Aβ peptide, we assayed α-secretase and β-secretase activity. Remarkably, TBI rats administrated with LV-ADAM10 showed an increase in α-secretase activity compared to the TBI group. Conversely, there was significant reduction in TBI rats treated with LV-siADAM10 (Figure 10F). Moreover, β-secretase activity was not affected when ADAM10 up-/down-regulation (Figure 10G). Additionally, we analyzed the protein expression levels of APP, Aβ_42_, αAPP (the product of a-secretase cleavage) and βAPP (the product of β-secretase cleavage) in the N2A cells by Western blot (Figure 10H). The results suggested that Aβ_42_ and βAPP protein levels were clearly increased in ADAM10 loss-of-function models and decreased in ADAM10 gain-of-function models (Figure 10I and 10J). Conversely, αAPP expression was enhanced by ADAM10 overexpression but ameliorated by ADAM10 knockdown in N2A cells exposed to glutamate (Figure 10K). However, there was no significant difference in APP levels. Together, the above findings indicated that the reduction in ADAM10 expression induced by a higher miR-144 levels post-injury is involved in cognitive dysfunction by directing APP processing toward the β-secretase to increase Aβ production.

### Elevated miR-144 promoted Aβ deposition-induced cognitive deficits by suppressing ADAM10 expression

**Figure 11:**
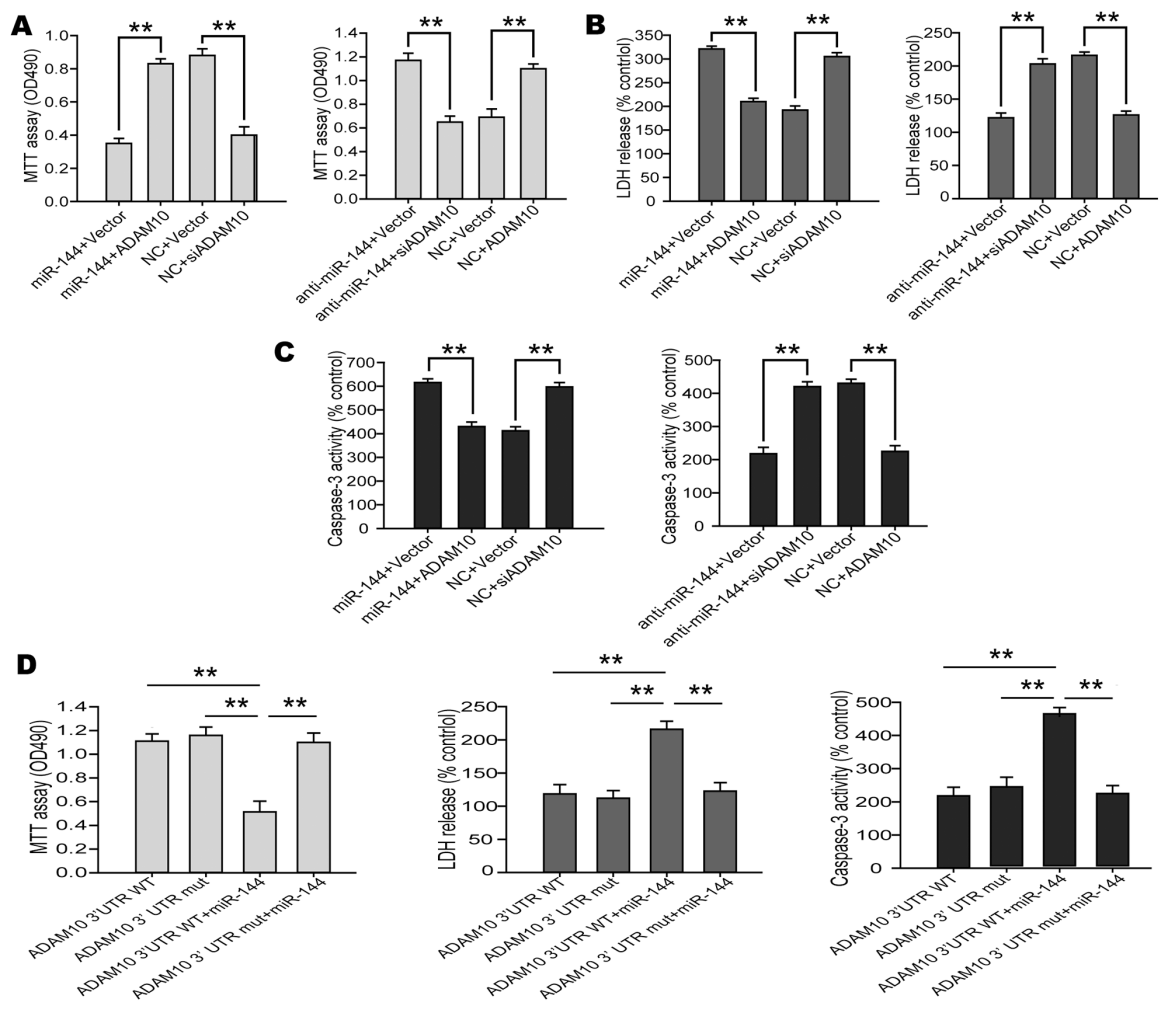
miR-144 involved in the regulation of TBI-induced brain injury by suppressing ADAM10 expression **(A-C)** Overexpression of ADAM10 blocked miR-144-induced neuron injury including cell viability **(A)**, LDH release **(B)** and caspase-3 activity **(C)** in the N2A cells, and the silencing of ADAM10 by siRNA mimicked the effects of miR-144. **(D)** miR-144 promoted TBI-induced brain injury though suppressing of ADAM10 expression. The data are showed as mean ± SD from three independent experiments.

**Figure 12:**
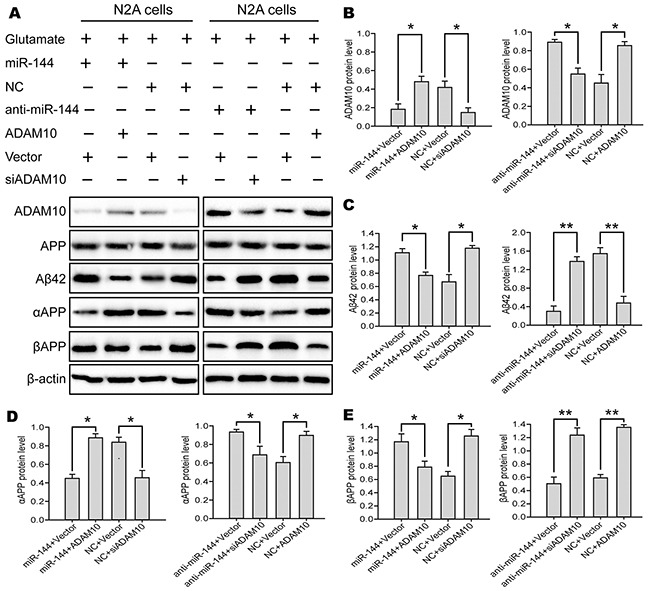
miR-144 involved in the regulation of TBI-induced cognitive impairments by suppressing ADAM10 expression **(A)** Representive bands from three independent Western blot analyses of ADAM10, APP, Aβ_42_, αAPP, and βAPP proteins in the N2A cells. **(B-E)** Quantitative date of these protein was assayed by densitometry. The data are showed as mean ± SD from three independent experiments. *P< 0.05, ^*^P < 0.01 versus the control group, ^#^*P*< 0.05, ^##^*P*< 0.01 versus the glutamate group.

The above findings prompted us to examine whether miR-144 induced cognitive impairments through suppression of ADAM10 signaling. For this purpose, we investigated the contribution of ADAM10 to the biological effects of miR-144 in Glu-induced neuron injury. ADAM10 siRNA and LV-anti-miR-144 (or LV-ADAM10 and LV-miR-144) were co-transfected into the N2A cells followed by treatmented with glutamate respectively. Interestingly, Cell viability (Figure 11A), LDH release (Figure 11B) and cell apoptosis (Figure 11C) assays showed that overexpression of ADAM10 blocked elevated miR-144-mediated cell injury. Conversely, silencing of ADAM10 mimicked the effects of miR-144. Silencing of ADAM10 by siRNA abrogated anti-miR-144 induced neuroprotective effects of miR-144 inhibitor in Glu-treated neurons. Furthermore, the ADAM10 with 3’UTR that contain the wild type (WT) potential binding site or that of mutant co-transfected with miR-144. I found that co-transfection ADAM10 3’UTR WT and miR-144 could reverse the effect of ADAM10 on cell injury, whereas, co-transfection ADAM10 3’UTR mut and miR-144 have no effects (Figure 11D). In addition, we also examined the protein levels of ADAM10, APP, Aβ_42_, αAPP, and βAPP. The results suggested that the effects of miR-144 on cognitive-related protein expression were antagonized and mimicked by overexpression and silencing of ADAM10, respectively. Meanwhile, the downregulation of Aβ_42_ and βAPP, as well as the upregulation of αAPP caused by miR-144 inhibition were effectively reversed by ADAM10 silencing (Figure 12). These results indicated that miR-144 promoted Aβ deposition involved in cognitive deficits by suppressing ADAM10 expression after TBI.

## DISCUSSION

TBI is a common cause for cognitive and communication problems, but little can be done to reverse TBI-induced cognitive dysfunction. For develop effective therapeutic strategies, more studies need to done to define the biological mechanisms underlying cognitive impairments. Previous evidences have demonstrated that TBI induced extensive molecular and biological alterations, including protein, mRNA and miRNA expression [39–41], which greatly affected pathophysiology and functional outcome after TBI. Especially, aberrant expressions of numerous miRNAs have been well identified in rats in response to TBI by miRNA microarray analysis and qRT-PCR, and considered as the key molecular targets for TBI treatment [9, 42, 43]. In addition, The number and expression of aberrant miRNAs at different time points varied greatly from 1 h to 7 d post-TBI, indicating that they may be used as molecular markers for evaluation of TBI progress [12]. Moreover, bioinformatics and gene ontology analyses revealed that some target genes of dysregulated miRNAs involved in TBI were associated with neuronal development, axonal regeneration, synaptic plasticity and cognitive function [44]. Therefore, the involvement of altered miRNAs in the pathological mechanism of TBI is highly complex and requires further study. Identification of key abnormally expressed miRNA after TBI will be helpful to further understand the potential mechanism, and regulation of its expression may be of therapeutic benefit.

miR-144, originally identified as an anti-oncogene and located in chromosome 10, played a crucial role in tumor cell proliferation, apoptosis, invasion, and metastasis [45]. Genome-wide analysis of miRNA expression has revealed that the sole miR-144 was consistently up-regulated in the aging brain, which is usually accompanied by a continuous cognitive decline [26]. The elevated expression of miR-144 increase the susceptibility to environmental stress and is considered a major risk factor for development of AD and other prevalent neurodegenerative diseases [28]. These evidences demonstrated that ectopic expression of miR-144 was strongly associated with cognitive disorder. However, whether miR-144 is involved in TBI-induced cognitive dysfunction remains unclear. In this study, we focused on investigating the potential functions and mechanisms for miR-144 in hippocampal alterations and cognitive impairments after TBI. We first found a significant increase in miR-144 expression in the plasma of patients subjected to TBI and a significant correlation was observed between miR-144 and GCS scores. As expected, down-regulation of miR-144 expression was also verified in a rat model of TBI *in vivo* and Glu-induced neuron injury model *in vitro*, consistent with previous reported [10]. Furthermore, the current study suggested consistent up-regulation of miR-144 in the hippocampal CA1 region at various time points up to 14 days post-TBI. Based on these finding, we hypothesized that miR-144 could play a critical role in the pathophysiological process of TBI, especially the development of TBI-induced cognitive dysfunction.

In order to evaluate the functional significance of miR-144 in TBI *in vivo*, miR-144 agomir or antagomir were administered via intracerebroventricular injection to rats suffered from TBI. We found that overexpression of miR-144 aggravated the prognosis of TBI, whereas inhibition of miR-144 provided neuroprotective effects against TBI, as demonstrated by a decrease in lesion volume, alleviation of brain edema, and an improvement in neurological function. More importantly, TBI-induced cognitive deficits were significantly ameliorated by miR-144 knockdown, as evidenced by improving spatial learning and memory ability, non-spatial hippocampus-mediated memory, and TBI-induced LTP impairment in NMDAR dependent manner. NMDAR is critically involved in hippocampus-dependent learning, hippocampal synaptic plasticity and memory encoding. Among, GluN2A subunit is required for rapidly acquired spatial working memory, and GluN2B is critical for a long-delay working memory task [46]. GluN2 mutants revealed distinct contributions from GluN2A- and GluN2B-containing NMDARs to rapidly and slowly acquired memory performance such as lacking bidirectional synaptic plasticity in the dentate gyrus and perform poorly on spatial pattern separation tasks [47]. Other studies have shown evidence for NMDAR dysfunction following experimental TBI and impaired induction of long-term potentiation up to 15 days post-injury [48]. *In vitro*, Glu-induced neuron injury was performed to mimic TBI-mediated neurons injury in the hippocampus of rats, followed by LV-miR-144/ LV-anti-miR-144 were infected to up-/down-regulate miR-144 expression. The results suggested that miR-144 knockdown could increase cell viability reduced LDH release and cell apoptosis, contributing to neuron injury recovery. Significantly, miR-144 inhibition reversed the increase in Aβ production and the reduction in NMDAR expression induced by glutamate, which closely related to cognitive and momery. In contrast, miR-144 overexpression accelerated Glu-induced neuron injury and cognitive-related proteins disorders. Taken together, these findings indicated that ectopic higher miR-144 levels participated in TBI-induced cognitive impairments, and it could be a potential therapeutic target for interventions.

In the present study, we further explored the molecular mechanisms underlying the neuroprotective effects of miR-144. Recent studies have uncovered that miR-144 plays a central coordinating role by targeting to ataxin 1 (ATXN1) gene in the aging brain at the post-transcriptional manner [26]. Here, biological information analysis showed that ADAM10, a gene encoding the α-secretase, was a putative target gene of miR-144, consistent with previous research [48]. Also, we found miR-144 expression was inversely correlated with ADAM10 protein level *in vivo* and *in vitro*. Importantly, our luciferase reporter assay demonstrated the miR-144 inhibited ADAM10 expression by directly binding to 3’-UTR region of ADAM10 mRNA, indicating that ADAM10 was direct downstream target gene of miR-144. Nevertheless, miR-144 dramatically decrease the protein expression of ADAM10, whereas have no effect on ADAM10 mRNA level, suggesting that the negative regulation of ADAM10 by miR-144 is mainly achieved at the translational level. Moreover, it was first observed that ADAM10 protein expression are down-regulated but ADAM10 mRNA level are up-regulated in the hippocampus and the N2A cells post-injury. These two seemingly incompatible results also indicated that translational regulation of ADAM10 by miR-144 existed in the pathogenesis of TBI. Additionally immunostaining and Western blot results further demonstrated overexpression of miR-144 remarkably suppressed protein expression of ADAM10, but didn't affect ADAM10 mRNA level. However, whether the neuroprotective effects of miR-144 was related to its negative regulation of ADAM10 protein remains unknown.

Increasing evidences supported that ADAM10 could prevent Aβ formation and hippocampal behavioral defects in AD mice [49]. Moreover, TBI, a major risk factor for Alzheimer-like dementia, could lead to Aβ peptides accumulating in the brain tissues, which was believed to be the main pathophysiological cause of TBI-induced cognitive decline and long-term neurodegeneration [50, 51]. It was reported that Aβ accumulation occurred with high frequency after TBI, particularly in injured axons of in the hippocampal neurons [52]. The production of Aβ peptides depended on cleavage pathway of the amyloid precursor protein (APP) encoding the neuronal membrane protein by the β-secretase and γ-secretase [53]. Interestingly, the Aβ peptides formation was avoided by an alternate APP cleavage pathway mediated by the α-secretase and γ-secretase [54]. Indeed, ADAM10, as a gene encoding α-secretase, suppressed Aβ production by directing APP processing toward the a-secretase [55]. Remarkably, ADAM10 is also known to induces activation of Noth pathway to repair neuronal damage and invoke nuclear gene for neurogenesis [56]. Here, to investigate effects of ADAM10 on Aβ peptides deposition-mediated cognitive deficits, we induced or silenced ADAM10 in TBI rats and N2A cells. The results indicated the induction of Aβ formation and cognitive deficits after TBI was mitigated in rats over-expressing ADAM10, and exacerbated in rats silencing ADAM10. Thus, we conclude that the reduction in ADAM10 by ectopic miR-144 is mechanistically linked to Aβ accumulation-induced cognitive deficits. Overexpression of ADAM10 appears to direct APP processing toward the a-secretase away from the β-secretase, which results in a reduction in Aβ production, further to improve cognitive dysfunction. More importantly, silenceing of ADAM10 by siRNA blocked the improvement of cognitive dysfunction caused by miR-144 inhibition. In contrast, overexpression of ADAM10 reversed cognitive deficits caused by miR-144. In conclusion, the above findings demonstrated that elevated expression of miR-144 contributed to TBI-induced cognitive deficits resulted from Aβ deposition by directly suppressing ADAM10 protein expression and inhibition of miR-144 conferred a better cognitive outcome.

## MATERIALS AND METHODS

### Blood samples from TBI patients

A total of 68 patients diagnosed with TBI according to clinical information and head computed tomography (CT) analysis were recruited in the Department of Neurosurgery, Beijing Tiantan Hospital of Capital Medical University (Beijing, Chnia), from January to December 2014. The inclusion criteria for all subjects included aged 18 to 50 years old, injury occurring within the preceding 24 h. For the GCS score when hospitalized, 21 cases were 13-15 scores, 24 cases were 9-12 scores, and 23 cases were 3-8 scores. The equivalent number of age-matched healthy subjects were gathered as control groups (n=20). Blood was collected from all subjects for assessments.

The study protocols were approved by the Ethics Committee on Human Research of Capital Medical University. All experiments were performed in accordance with the relevant guidelines and regulations, approved by this Committee. The written informed consent was obtained from each participant in the study.

### Animal model of TBI

All experimental procedures were carried out in accordance with the guidelines of the Chinese Council on Animal Protection, and approved by the Ethics Committee of Capital Medical University for the use of animals in this study. A total of 360 male Sprague-Dawley (SD) rats (aged 12 weeks and weighing 350–375 g) were purchased from Vital River Laboratory Animal Technology Co. Ltd. (Vital River, Beijing, China). All the animals were housed with a standard of 12 h light/dark cycle and free access to water and food before and after operation. The rat model of TBI was induced by using a modified weight-drop device, as described previously by Marmarou [57]. Briefly, the rats were anesthetized with sodium pentobarbital (Nembutal 60 mg/kg). After a midline incision was made to expose the skull between bregma and lambda suture lines, a steel disc (10 mm in diameter and 3 mm in thickness) was adhered to the skull using dental acrylic. Animals were moved onto a foam mattress underneath a weight-drop device where a weight of 450 g falls freely through a vertical tube from 1.5 m onto the steel disc. Sham-operated animals underwent the same surgical procedure without weight-drop impact. Rats were housed in individual cages after surgery and placed on heat pads (37°C) for 24 h to maintain normal body temperature during the recovery periods.

### Experimental groups and intracerebroventricular injection

All rats were randomly divided into six groups: sham group, TBI group, miR-144 agomir (agomir), agomir negative control (agomir NC), miR-144 antagomir (antagomir), and antagomir negative control (antagomir NC). The sequences were as follows: miR-144 agomir: 5′-UACAGUAUAGAUGAUGUACU-3′, agomir negative control: 5′-UUCUCCGAACGUGUCACGUTT-3′, miR-144 antagomir: 5′-AGUACAUCAUCUAUACUGUA-3′, antagomir negative control: 5′-CAGUACUUUUGUGUAGUACAA-3′ (GeneChem, Shanghai, China). miRNA oligomers (5μL) were then combined with lipofectamine-2000 (12.5 μL) (Invitrogen, Carlsbad, CA, USA) in an RNase-free PCR tube and incubated for 30 min at room temperature. At 15 min after TBI, rats received a single intracerebroventricular injection of miR-144 agomir, miR-144 antagomir, or their corresponding negative control as previously reported [42]. In addition, in LV-ADAM10, LV-NC, LV- siADAM10 or LV-siNC group, rats received a single intracerebroventricular injection of lentivirus particles (1.0μL) respectively at three days before TBI induction. The Hamilton brain infusion syringe was stereotaxically injected into the right lateral ventricle (coordinates: 1.5 mm caudal to bregma; 1.1 mm lateral to midline; 4.5 mm deep from the surface of skull) at the rate of 1μL/min.

### Modified neurological severity score (mNSS) test

Posttraumatic neurological impairments were assessed using mNSS test at 1 d, 3 d, 7 d and 14 days post-TBI, Assessments in the neuroscore include motor, sensory, reflex, and balance tests, as described previously [58]. The test was performed by the same observer who was blinded to the experimental conditions and treatments. Neurological function was graded on a scale of 0–18, where a total score of 18 points indicates severe neurological deficit and a score of 0 indicates normal performance. 13–18 indicates severe injury, 7–12 indicates mean-moderate injury, and 1–6 indicates mild injury.

### Evaluation of brain water content

The brain water content (BWC) was measured using the wet-dry weight method as our previously described [59]. Briefly, rats were anesthetized with chloral hydrate (30 ml/kg, i.p.) and sacrificed to remove the brain at 3 d post-injury. The brain tissues were weighed immediately to obtain the wet weight (ww) and then dried in an electric oven at 100°C for 24 h to determine the dry weight (dw). The BWC was calculated as follows: (ww - dw) / ww × 100%.

### Morris water maze test

The hippocampus-dependent spatial learning and memory was assessed using the morris water maze (MWM) task from 14 d to 18 d following TBI as described previously [60]. Rats were trained to find the platform before operation. For each trial, the rats were randomly placed into a quadrant start points (N, S, E or W) facing the wall of the pool and allowed a maximum of 60 s to escape to the platform, rats which failed to escape within 90 s were placed on the platform for a maximum of 20 s and returned to the cage for a new trial. In this phase, each animal was tested for three trials per day for six consecutive days. Maze performance was recorded by a video camera suspended above the maze and interfaced with a video tracking system (HVS Imaging, Hampton, UK). The average escape latency of a total of six trials, time spent in goal quadrant, and average swim speed was calculated.

### Novel object recognition test

Novel object recognition (NOR) test was used to evaluate non-spatial hippocampus-mediated memory as previously described [61]. The NOR tests were carried out on a day before injury and 18 d post-trauma. This task was performed six times to evaluate functional changes in cognition over time, based on the innate tendency to explore new objects within their environment. The time the animals spent on exploring the familiar and novel objects was recorded in this test.

### Electrophysiological recordings

Tungsten stimulating electrodes were placed on the Schaffer collaterals in the CA3 subregion of hippocampal slices. Input–output curves were generated at the beginning of each experiment to determine maximal and half-maximal responses and voltage settings (maximum response was also determined at the end of the experiment). The voltage was then set to evoke an EPSP response that was approximately half-maximal in amplitude. To evoke orthodromic field excitatory postsynaptic potentials (fEPSPs or EPSPs), monophasic test pulses were delivered to the slice every 60 seconds (Grass S48 stimulator, War-wick, RI) with a stimulus duration of 0.1 ms. Recording was accomplished with an AxoClamp 2B amplifier (Axon Instr., Foster City, CA) in continuous current clamp bridge mode. EPSPs were obtained using glass microelectrodes (2.3 Mohms) filled with recording buffer, which were generated in a standard recording chamber - submerged configuration (RC-27L, Warner Inst., Hamden, CT) at 31-32°C. EPSP responses were recorded from the CA1 dendriticarbor. EPSP slope values were calculated by measuring the rise/run (i.e., 10-90% of trace). Responses were amplified (gain 50x) low-pass filtered at 6 kHz and digitized (20 kHz) (DIGIDATA 1322A, Axon Instr., Foster City, CA). For elicitation of LTP, after 30 min of stable baseline recording of fEPSPs evoked at the current intensity that triggered 50% of the maximal fEPSP response, theta burst stimulation (TBS, 4 pulses at 100 Hz) was applied at 200ms intervals to elicite LTP, then continued for 60 min with stimulation of fEPSPs every 1min. Data were acquired with Clampex 9.2(Axon Instr., Foster City, CA), analyzed initially with Clampfit 10.0 (Axon Instr., Foster City, CA).

### Primary hippocampal neuron, HT22 and N2A cells culture

Primary cultures of hippocampal neurons were prepared and maintained as previously described [62]. Briefly, the hippocampus were obtained from SD rats at 18 days of gestation (E18), and treated with papain (100 mg/ml, Worthington, Lakewood, NJ, USA) for 10 min at 37 °C. Cell suspension was then centrifuged at 1000 rpm for 5 min, Dissociated neurons were plated at a density of 20,000–30,000 cells/cm^2^in clear-bottom, black-walled 96-well amine plates (BD Biosciences, Auckland, New Zealand), and maintained in a humidified atmosphere of 5 % CO_2_ at 37 °C, with Neurobasal medium (Gibco, NY, USA)) supplemented with 2% B27 (Gibco, NY, USA), 200 mM GlutaMax (Invitrogen, Carlsbad, CA, USA), and 1% penicillin/streptomycin (Gibco, NY, USA). After 24 h, one-half of the medium was replaced with fresh culture medium added cytosine-β-D-arabinofuranoside (Ara-C, Sigma, St. Louis, MO, USA) at a final concentration of 5μmol/L for 48 h, so as to inhibit glial cell proliferation. The HT22 cells, a murine hippocampal cell line, and N2A cells, a mouse neuroblastoma N2a cells, were purchased from the Shanghai Baili Biology Company (Shanghai, China). Both HT22 and N2A cells were cultured in DMEM (Gibco, NY, USA) supplemented with 10 % FBS (Gibco, NY, USA) and 1 % penicillin/streptomycin. The cells were incubated for 24 h prior to experimental treatments.

### Lentiviral vector construction and infection

The full length miR-144, miR-144-specific inhibitor or their corresponding negative control oligonucleotide were cloned into the GV280 Lentiviral vector (Genechem, Shanghai, China) containing a CMV-driven enhanced green fluorescent protein (EGFP) reporter, to construct lentivirus encoding miR-144 (LV-miR-144), lentivirus inhibiting miR-144 (LV-anti-miR-144), LV-NC and LV-anti-NC. Cells under good culture conditions from each experimental group were seeded into 6-well plates at a density of 5 × 10^4^ cells/well one day prior to viral infection, and were infected with LV-miR-144, LV-NC, LV-anti-miR-144, LV-anti-NC, respectively. Three days after infection, the expression of EGFP was monitored under a fluorescence microscope (Leica, Solms, Germany) and miR-144 levels were detected by real-time PCR to assess infection efficiency.

### Cell viability, LDH release and caspase-3 activity assay

Cell viability was assessed using 3-(4,5-dimethylthiazol-2-yl)-2,5 diphenyl tetrazolium bromide (MTT) assay. MTT assay was performed at 3 h, 6 h, 12 h, 24 h, and 48 h after infection. Briefly, the cultured cells were seeded in 96-well culture plates at a density of 4×10^3^ cells/well and incubated with 0.5 mg/ml MTT solution (Sigma, St. Louis, MO, USA). Then, the cells were treated with 0.1% DMSO (Sigma, St. Louis, MO, USA) in order to dissolve the formazan crystals. The absorbance was measured at 490 nm using a SpectraMax 190 spectrophotometer (Molecular Devices, Sunnyvale, CA, USA). Neuronal injury was assessed by quantitative measurement of LDH in the culture medium. LDH release was measured through the LDH Kit (Jiancheng Bioengineering Institute, Nanjing, China) and quantitated by measuring the absorbance at 490 nm. The caspase-3 activity was measured using caspase-3 activity assay kit (Beyotime, Shanghai, China). All results were normalized against control group.

### Luciferase reporter assays

Three online prediction programs, TargetScan, miRanda, and PicTar, were used to identify candidate targets for miR-144. The gene for ADAM10, predicted by all three databases and associated with cognitive function, was further studied as a potential target. Sequence of segments with WT or mutant 3’-UTR region of ADAM10 were synthesized and cloned into the pGL3 vector (GeneChem, Shanghai, China). All constructs were verified by sequencing. First, the N2A or HEK293 cells were seeded at 0.5×10^5^ cell per well in 24-well plates 24 h prior to transfection. The following day, the pGL3 vector containing WT 3’-UTR of ADAM10 mRNA or mutant forms was co-transfected with LV-miR-144 into N2A or HEK293 cells. After 48 h, all cells were harvested according to manufacturer's protocol (Promega, Madison, WI), and the Firefly and Renilla luciferase activity were determined using dul-luciferase reporter assay system (Promega, Madison, WI) with a Victor X machine (PerkinElmer, Boston, MA). Firefly luciferase activity was normalized to Renilla luciferase activity. Three independent experiments were performed in triplicate.

### RNA extraction and real-time PCR assays for miRNA and mRNA

Total RNA was extracted from the plasma, brain tissue or cultured cells with TRIzol Reagent (Invitrogen, Carlsbad, CA, USA). Reverse transcription reactions were performed with the Superscript First Strand cDNA synthesis system (Invitrogen, Carlsbad, CA, USA). According to the manufacturer's instructions. Real-time PCR was performed with One Step SYBR® Prime Script TM RT-PCR Kit II (Takara, Japan). PCR was performed using the following primers: miR-144 forward, 5-GGGGGGGGGGGGGTACAGTATAGATGATGTACTAA-3, and U6 forward, 5-GCAAGGATGACACGCAAATTCGT-3. ADAM10 forward, 5-TCGAACCATCACCCTGCAACCT-3 and reverse, 5-GCCCACCAATGAGCCACAATCC-3. GAPDH: forward, 5’-CAAAGTTGTCATGGATGACC-3’, reverse, 5’-CCATGGAGAAGGCTGGGG-3’. The U6 and GAPDH served as an internal control for miRNA and mRNAs assays. The cycle threshold (Ct value) was acquired using the Opticon Monitor Analysis Software (MJ Research, St. Bruno, Quebec, Canada). The data were analyzed using 2^-ΔΔCt^ method and the values were presented by relative quantity.

### Combined miRNA ISH and IF staining

The biomarkers of neurons (NeuN) and astrocytes (GFAP) were respectively counterstained with miR-144 using the miRNA ISH kit (Boster, Wuhan, China). the paraffin sections were dewaxed, rehydrated and retrieved, and then underwent pretreatment with standard saline citrate (SSC) for 25 min. Pre-hybridization was performed with pre-hybridized solution for 2 h at 40°C. Next, The sections were incubated with locked nucleic acid-modified miR-144 probe. Thereafter, sections were blocked using 3% BSA for 30 min at 37°C and incubated overnight at 4°C with the primary antibody of the above mentioned biomarkers. They were then incubated with a mixed solution of the secondary antibody and biotinylated anti-digoxin antibody for 1 h at 37°C, The nuclei were counterstained with DAPI.

### ELISA analysis

The concentration of Aβ_42_ in the cultural supernatant of N2A cells was detected Enzyme-Linked ImmunoSorbent Assay (ELISA) analyses (R&D Systems, Minneapolis, Minnesota, USA) according to the manufacturer's instructions. The absorbance was read at 490 nm on an ELISA plate scanner (Molecular Devices, Sunnyvale, CA, USA). All experiments are performed at least in triplicate samples and results are presented as the mean value.

### Immunohistochemistry staining

After the experimental protocol, rats were anesthetized and sacrificed and the brain tissues were removed for immunohistochemistry staining. The immunohistochemistry analysis performed in accordance with the instructions of SABC immunohistochemistry kit (Beyotime, Shanghai, China). The sections were incubated in 5%BSA solution for 20 min, followed by the microwave antigen retrieval and inactivation with 3%H_2_O_2_ procedures. Subsequently, sections were incubated overnight at 4°C with rabbit anti-Aβ_42_ or rabbit anti-caspase 3 polyclonal antibodies (Santa Cruz Biotechnology, Santa Cruz, CA, USA), and then with horseradish peroxidase-conjugated anti-rabbit IgG for 60 min. Diaminobenzidine (Beyotime, Shanghai, China) was used to reveal the immunohistochemical reaction.

### Immunofluorescence staining

The frozen sections were treated with 0.4 % Triton-100 for 20 min, and blocked in normal donkey serum for 1 h. For double labeling, sections were incubated with a mixture of rabbit anti-ADAM10 polyclonal antibody (diluted 1:50) and mouse anti-NeuN (Millipore, CA, USA, diluted 1:50) overnight at 4°C. The next day, the sections were incubated with a mixture of fluorescein-conjugated anti-rabbit IgG and anti-mouse IgG (Santa Cruz Biotechnology, Santa Cruz, CA, USA, diluted 1:1,000) for 2 h at 37°C in the dark. The nuclei were counterstained with DAPI. Photographs were taken in a laser scanning confocal microscope (OLYMPUS FV1000).

### Western blot analysis

Hippocampal tissues and cultured cells were homogenized on ice in ice-cold lysis buffer containing 137 mM NaCl, 20 mM Tris-HCl (pH 8.0), 10% glycerol, 1% NP-40, 10 mg/mL aprotinin, 1 mM PMSF, 1 mg/ mL leupeptin, and 0.5 mM sodium vanadate, and processed for western blot as previously described [63]. The protein concentration was determined by the BCA reagent (Solarbio, Beijing, China) method. Samples (20μg of protein) were subjected to sodium dodecyl sulfate polyacrylamide gel electrophoresis (SDS-PAGE). Separated proteins on the gel were transferred onto PVDF membranes (Roche Diagnostics, Mannheim, Germany). Blots were blocked with 5 % fat-free dry milk for 1 h at room temperature and then incubated with primary antibodies overnight at 4 °C, including rabbit anti-NMDAR, ADAM10, APP, Aβ_42_, αAPP, βAPP, and β-actin polyclonal antibody (Santa Cruz Biotechnology, Santa Cruz, CA, USA, diluted 1:500). Subsequently, the blots were thoroughly washed and incubated with horseradish peroxidase-conjugated secondary antibodies (Cell Signaling Technology, Inc., Danvers, MA, USA, diluted 1:6,000) for 2 h at room temperature. The immunoblot on the membrane was visible after developing with an enhanced chemiluminescence (ECL) detection system and results were analyzed with National Institutes of Health Image 1.41 software (Bethesda, MD, USA).

### Statistical analysis

All experiments were repeated three times and similar results were obtained. Statistical analysis was performed using the SPSS 16.0 statistics software (SPSS, Chicago, IL). Data were expressed as mean ±standard deviation (SD). Statistical analysis was performed using ANOVA and followed by the Student-Newman-Keuls post hoc tests or Student't test (two means comparison). *P* value of less than 0.05 was considered statistically significant.

## References

[1] Hemphill MA, Dauth S, Yu CJ, Dabiri BE, Parker KK (2015). Traumatic brain injury and the neuronal microenvironment: a potential role for neuropathological mechanotransduction. Neuron.

[2] Feigin VL, Theadom A, Barker-Collo S, Starkey NJ, McPherson K, Kahan M, Dowell A, Brown P, Parag V, Kydd R, Jones K, Jones A, Ameratunga S, BIONIC Study Group (2013). Incidence of traumatic brain injury in New Zealand: a population-based study. Lancet Neurol.

[3] Sun L, Gao J, Zhao M, Jing X, Cui Y, Xu X, Wang K, Zhang W, Cui J (2014). The effects of BMSCs transplantation on autophagy by CX43 in the hippocampus following traumatic brain injury in rats. Neurol Sci.

[4] Blennow K, Hardy J, Zetterberg H (2012). The neuropathology and neurobiology of traumatic brain injury. Neuron.

[5] Gatson J, Liu MM, Wigginton JG, Wolf S, Minei JP (2012). Resveratrol decreases inflammation in the hippocampus of mice suffering from moderate TBI. Circulation.

[6] Tian-Hao B, Wei M, Jian-Hong H, Mei Y, Yong Y, Wei-Wei W, Yu-Hong Z (2014). Spontaneous running wheel improves cognitive functions of mouse associated with miRNA expressional alteration in hippocampus following traumatic brain injury. J Mol Neurosci.

[7] Hu T, Zhou FJ, Chang YF, Li YS, Liu GC, Hong Y, Chen HL, Xiyang YB, Bao TH (2015). miR21 is associated with the cognitive improvement following voluntary running wheel exercise in TBI mice. J Mol Neurosci.

[8] Marciano PG, Brettschneider J, Manduchi E, Davis JE, Eastman S, Raghupathi R, Saatman KE, Speed TP, Stoeckert CJ, Eberwine JH, McIntosh TK (2004). Neuron-specific mRNA complexity responses during hippocampal apoptosis after traumatic brain injury. J Neurosci.

[9] Sabirzhanov B, Stoica BA, Zhao Z, Loane DJ, Wu J, Dorsey SG, Faden AI (2016). miR-711 upregulation induces neuronal cell death after traumatic brain injury. Cell Death Differ.

[10] Liu L, Sun T, Liu Z, Chen X, Zhao L, Qu G, Li Q (2014). Traumatic brain injury dysregulates microRNAs to modulate cell signaling in rat hippocampus. PLoS One.

[11] Sun TY, Chen XR, Liu ZL, Zhao LL, Jiang YX, Qu GQ, Wang RS, Huang SZ, Liu L (2014). Expression profiling of microRNAs in hippocampus of rats following traumatic brain injury. J Huazhong Univ Sci Technolog Med Sci.

[12] Sharma A, Chandran R, Barry ES, Bhomia M, Hutchison MA, Balakathiresan NS, Grunberg NE, Maheshwari RK (2014). Identification of serum microRNA signatures for diagnosis of mild traumatic brain injury in a closed head injury model. PLoS One.

[13] Wang WX, Visavadiya NP, Pandya JD, Nelson PT, Sullivan PG, Springer JE (2015). Mitochondria-associated microRNAs in rat hippocampus following traumatic brain injury. Exp Neurol.

[14] Bartel DP (2009). MicroRNAs: target recognition and regulatory functions. Cell.

[15] Kosik KS (2010). MicroRNAs and cellular phenotypy. Cell.

[16] Friedman RC, Farh KK, Burge CB, Bartel DP (2009). Most mammalian mRNAs are conserved targets of microRNAs. Genome Res.

[17] Ambros V (2003). MicroRNA pathways in flies and worms: growth, death, fat, stress, and timing. Cell.

[18] Cohen SM (2009). Use of microRNA sponges to explore tissue-specific microRNA functions in vivo. Nat Methods.

[19] Sempere LF, Freemantle S, Pitha-Rowe I, Moss E, Dmitrovsky E, Ambros V (2004). Expression profiling of mammalian microRNAs uncovers a subset of brain-expressed microRNAs with possible roles in murine and human neuronal differentiation. Genome Biol.

[20] Sun E, Shi Y (2015). MicroRNAs: small molecules with big roles in neurodevelopment and diseases. Exp Neurol.

[21] Ouyang YB, Xu L, Yue S, Liu S, Giffard RG (2014). Neuroprotection by astrocytes in brain ischemia: importance of microRNAs. Neurosci Lett.

[22] Goedeke L, Fernández-Hernando C (2014). MicroRNAs: a connection between cholesterol metabolism and neurodegeneration. Neurobiol Dis.

[23] Di Y, Lei Y, Yu F, Changfeng F, Song W, Xuming M (2014). MicroRNAs expression and function in cerebral ischemia reperfusion injury. J Mol Neurosci.

[24] Codocedo JF, Ríos JA, Godoy JA, Inestrosa NC (2016). Are microRNAs the molecular link between metabolic syndrome and Alzheimer's disease?. Mol Neurobiol.

[25] Meissner L, Gallozzi M, Balbi M, Schwarzmaier S, Tiedt S, Terpolilli NA, Plesnila N (2016). Temporal profile of microRNA expression in contused cortex after traumatic brain injury in mice. J Neurotrauma.

[26] Persengiev S, Kondova I, Otting N, Koeppen AH, Bontrop RE (2011). Genome-wide analysis of miRNA expression reveals a potential role for miR-144 in brain aging and spinocerebellar ataxia pathogenesis. Neurobiol Aging.

[27] Li W, Chen L, Li W, Qu X, He W, He Y, Feng C, Jia X, Zhou Y, Lv J, Liang B, Chen B, Jiang J (2013). Unraveling the characteristics of microRNA regulation in the developmental and aging process of the human brain. BMC Med Genomics.

[28] Cheng C, Li W, Zhang Z, Yoshimura S, Hao Q, Zhang C, Wang Z (2013). MicroRNA-144 is regulated by activator protein-1 (AP-1) and decreases expression of Alzheimer disease-related a disintegrin and metalloprotease 10 (ADAM10). J Biol Chem.

[29] Lan F, Yu H, Hu M, Xia T, Yue X (2015). miR-144-3p exerts anti-tumor effects in glioblastoma by targeting c-Met. J Neurochem.

[30] Cai H, Xue Y, Wang P, Wang Z, Li Z, Hu Y, Li Z, Shang X, Liu Y (2015). The long noncoding RNA TUG1 regulates blood-tumor barrier permeability by targeting miR-144. Oncotarget.

[31] Lee ST, Kim M (2011). MicroRNAs in experimental models of movement disorders. J Mov Disord.

[32] Sachser RM, Santana F, Crestani AP, Lunardi P, Pedraza LK, Quillfeldt JA, Hardt O, Alvares LO (2016). Forgetting of long-term memory requires activation of NMDA receptors, L-type voltage-dependent Ca2+ channels, and calcineurin. Sci Rep.

[33] Sun L, Gao J, Zhao M, Cui J, Li Y, Yang X, Jing X, Wu Z (2015). A novel cognitive impairment mechanism that astrocytic p-connexin 43 promotes neuronic autophagy via activation of P2×7R and down-regulation of GLT-1 expression in the hippocampus following traumatic brain injury in rats. Behav Brain Res.

[34] Rahn KA, Slusher BS, Kaplin AI (2012). Glutamate in CNS neurodegeneration and cognition and its regulation by GCPII inhibition. Curr Med Chem.

[35] Nicholls RE, Sontag JM, Zhang H, Staniszewski A, Yan S, Kim CY, Yim M, Woodruff CM, Arning E, Wasek B, Yin D, Bottiglieri T, Sontag E (2016). PP2A methylation controls sensitivity and resistance to β-amyloid-induced cognitive and electrophysiological impairments. Proc Natl Acad Sci USA.

[36] Rodrigue KM, Kennedy KM, Devous MD, Rieck JR, Hebrank AC, Diaz-Arrastia R, Mathews D, Park DC (2012). β-Amyloid burden in healthy aging: regional distribution and cognitive consequences. Neurology.

[37] Kumar A, Foster TC (2013). Linking redox regulation of NMDAR synaptic function to cognitive decline during aging. J Neurosci.

[38] Kapai NA, Bukanova JV, Solntseva EI, Skrebitsky VG (2012). Donepezil in a narrow concentration range augments control and impaired by beta-amyloid peptide hippocampal LTP in NMDAR-independent manner. Cell Mol Neurobiol.

[39] Hu Z, Yu D, Almeida-Suhett C, Tu K, Marini AM, Eiden L, Braga MF, Zhu J, Li Z (2012). Expression of miRNAs and their cooperative regulation of the pathophysiology in traumatic brain injury. PLoS One.

[40] Redell JB, Liu Y, Dash PK (2009). Traumatic brain injury alters expression of hippocampal microRNAs: potential regulators of multiple pathophysiological processes. J Neurosci Res.

[41] Lei P, Li Y, Chen X, Yang S, Zhang J (2009). Microarray based analysis of microRNA expression in rat cerebral cortex after traumatic brain injury. Brain Res.

[42] Ge XT, Lei P, Wang HC, Zhang AL, Han ZL, Chen X, Li SH, Jiang RC, Kang CS, Zhang JN (2014). miR-21 improves the neurological outcome after traumatic brain injury in rats. Sci Rep.

[43] Sabirzhanov B, Zhao Z, Stoica BA, Loane DJ, Wu J, Borroto C, Dorsey SG, Faden AI (2014). Downregulation of miR-23a and miR-27a following experimental traumatic brain injury induces neuronal cell death through activation of proapoptotic Bcl-2 proteins. J Neurosci.

[44] Wang WX, Sullivan PG, Springer JE (2017). Mitochondria and microRNA crosstalk in traumatic brain injury. Prog Neuropsychopharmacol Biol Psychiatry.

[45] Gao F, Wang T, Zhang Z, Wang R, Guo Y, Liu J (2015). Regulation of activating protein-4-associated metastases of non-small cell lung cancer cells by miR-144. Tumour Biol.

[46] Zhang XH, Liu SS, Yi F, Zhuo M, Li BM (2013). Delay-dependent impairment of spatial working memory with inhibition of NR2B-containing NMDA receptors in hippocampal CA1 region of rats. Mol Brain.

[47] Kannangara TS, Eadie BD, Bostrom CA, Morch K, Brocardo PS, Christie BR (2015). GluN2A−/− mice lack bidirectional synaptic plasticity in the dentate gyrus and perform poorly on spatial pattern separation tasks. Cereb Cortex.

[48] Giza CC, Maria NS, Hovda DA (2006). N-methyl-D-aspartate receptor subunit changes after traumatic injury to the developing brain. J Neurotrauma.

[49] Gizem D, Diana W, Cohen DE, Leonard G (2014). Retraction notice to: SIRT1 suppresses β-amyloid production by activating the α-secretase gene ADAM10. Cell.

[50] Marklund N, Farrokhnia N, Hånell A, Vanmechelen E, Enblad P, Zetterberg H, Blennow K, Hillered L (2014). Monitoring of β-amyloid dynamics after human traumatic brain injury. J Neurotrauma.

[51] Gatson JW, Warren V, Abdelfattah K, Wolf S, Hynan LS, Moore C, Diaz-Arrastia R, Minei JP, Madden C, Wigginton JG (2013). Detection of β-amyloid oligomers as a predictor of neurological outcome after brain injury. J Neurosurg.

[52] Scott G, Ramlackhansingh AF, Edison P, Hellyer P, Cole J, Veronese M, Leech R, Greenwood RJ, Turkheimer FE, Gentleman SM, Heckemann RA, Matthews PM, Brooks DJ, Sharp DJ (2016). Amyloid pathology and axonal injury after brain trauma. Neurology.

[53] Quon D, Wang Y, Catalano R, Scardina JM, Murakami K, Cordell B (1991). Formation of β-amyloid protein deposits in brains of transgenic mice. Nature.

[54] Postina R, Schroeder A, Dewachter I, Bohl J, Schmitt U, Kojro E, Prinzen C, Endres K, Hiemke C, Blessing M, Flamez P, Dequenne A, Godaux E (2004). A disintegrin-metalloproteinase prevents amyloid plaque formation and hippocampal defects in an Alzheimer disease mouse model. J Clin Invest.

[55] Obregon DF, Rezai-Zadeh K, Bai Y, Sun N, Hou H, Ehrhart J, Zeng J, Mori T, Arendash GW, Shytle D, Town T, Tan J (2006). ADAM10 activation is required for green tea (-)-epigallocatechin-3-gallate-induced alpha-secretase cleavage of amyloid precursor protein. J Biol Chem.

[56] Hartmann D, Tournoy J, Saftig P, Annaert W, De Strooper B (2001). Implication of APP secretases in notch signaling. J Mol Neurosci.

[57] Marmarou A, Foda MA, van den Brink W, Campbell J, Kita H, Demetriadou K (1994). A new model of diffuse brain injury in rats Part I: pathophysiology and biomechanics. J Neurosurg.

[58] Zhang R, Liu Y, Yan K, Chen L, Chen XR, Li P, Chen FF, Jiang XD (2013). Anti-inflammatory and immunomodulatory mechanisms of mesenchymal stem cell transplantation in experimental traumatic brain injury. J Neuroinflammation.

[59] Cui C, Cui Y, Gao J, Sun L, Wang Y, Wang K, Li R, Tian Y, Song S, Cui J (2014). Neuroprotective effect of ceftriaxone in a rat model of traumatic brain injury. Neurol Sci.

[60] Hui-guo L, Kui L, Yan-ning Z, Yong-jian X (2010). Apocynin attenuate spatial learning deficits and oxidative responses to intermittent hypoxia. Sleep Med.

[61] Munyon C, Eakin KC, Sweet JA, Miller JP (2014). Decreased bursting and novel object-specific cell firing in the hippocampus after mild traumatic brain injury. Brain Res.

[62] Kim KM, Vicenty J, Palmore GT (2013). The potential of apolipoprotein E4 to act as a substrate for primary cultures of hippocampal neurons. Biomaterials.

[63] Sun LQ, Gao JL, Cui Y, Zhao MM, Jing XB, Li R, Tian YX, Cui JZ, Wu ZX (2015). Neuronic autophagy contributes to p-connexin 43 degradation in hippocampal astrocytes following traumatic brain injury in rats. Mol Med Rep.

